# Intellectual quotient score comparison between HIV-infected and HIV exposed children at the Peruvian National Institute of Child Health, Lima Peru

**DOI:** 10.1186/1742-4690-9-S1-P142

**Published:** 2012-05-25

**Authors:** Peggy C Martinez, Silvia M Montano, Julio Flores, Viviana Granados, Jose Rodriguez

**Affiliations:** 1 Instituto Nacional de Ciencias Neurologicas, Lima, Peru; 2 U.S. Naval Medical Research Unit-6 Lenka Kolevick; 3 Instituto Nacional de Salud del Nino, Lima, Peru

## Introduction

Pediatric HIV encephalopathy includes motor and cognitive deficits and can result in poor school performance, borderline intelligence or mental retardation. We compared intellectual quotient (IQ) between HIV-infected children and a group of HIV-negative children at the National Institute of Child Health in Peru. In addition, we studied the relationship between verbal and executive function and the clinical stage of HIV infection (CDC classification), HIV viral load, CD4 count and other potential risk factors.

## Materials and methods

We evaluated 28 HIV-infected children and 28 HIV-uninfected children matched by age and sex. The second group was exposed to HIV during pregnancy. Neuropsychologic testing to determine the intellectual quotient (IQ) included the Wechsler-Revised (WPPSI-R) for children between 3 - 7 years and 3 months, and the Wechsler Third Edition (WISC-III) for children older than 7 years and 4 months. Clinical records were reviewed to gather medical history, clinical and demographic data. T-test and Pearson’s correlation (R) were used to compare groups. IQ score was categorized as average or above average, low average, and borderline intellectual function or below for IQ scores > 90, 80-89, < 80, respectively.

## Results

The average IQ among HIV positive children was lower than the control Group (84.6 vs. 91.7, p =0.05). This difference was driven by the verbal sub test (81.2 vs. 90.3, p =0.05). The percentage of children with average IQ or higher was higher among the control group (57.1%) in comparison with the cases (32.1%); more HIV-infected children scored in the borderline intellectual function or below range (35.7% vs. 17.9%, respectively), but these differences were not statistically significant.

Children with advanced HIV infection had lower IQ scores and verbal performance than children with less-advanced HIV infection, but these differences were not statistically significant. Verbal test scores were negatively correlated with HIV viral load (R=- 0.424, p=0.024).

## Conclusions

HIV positive children show lower IQ scores in comparison with HIV negative children. Further studies are needed to confirm our findings.

